# Eriodictyol in Cancer Therapy: Reviewing Mechanistic Insights and Translational Opportunities

**DOI:** 10.3390/ijms27072924

**Published:** 2026-03-24

**Authors:** Sohail Mumtaz, Juie Nahushkumar Rana, Kainat Gul

**Affiliations:** 1 Department of Chemical and Biological Engineering, Gachon University, 1342 Seongnamdaero, Sujeong-gu, Seongnam-si 13120, Republic of Korea; 2 Fels Cancer Institute for Personalized Medicine, Lewis Katz School of Medicine at Temple University, Philadelphia, PA 19140, USA; 3 Department of Botany, Hazara University, Mansehra 21300, Pakistan

**Keywords:** eriodictyol, cancer signaling pathways, nanomedicine and drug delivery, apoptosis and ferroptosis, combination therapy

## Abstract

Eriodictyol, a naturally occurring flavanone, has appeared as a biologically versatile compound with increasing relevance in biomedical research, especially in cancers. Evidence over the past few decades indicates that eriodictyol influences cancer cell fate through coordinated modulation of cell-cycle control, survival, and regulated cell death pathways. Eriodictyol appears to reshape oncogenic signaling networks, including PI3K/Akt/mTOR and associated kinase cascades, thereby restricting proliferative capacity and lowering resistance thresholds. Studies consistently report cell-cycle arrest at critical checkpoints, accompanied by activation of both mitochondrial- and death-receptor-mediated apoptotic pathways through disruption of BCL-2 family balance, caspase engagement, and mitochondrial destabilization. Furthermore, eriodictyol alters intracellular redox dynamics in a dose-dependent manner, selectively sensitizing cancer cells to oxidative and metabolic stress. More recent findings extend its significance to inflammation-driven tumor progression and to the regulation of ferroptosis. Beyond intrinsic pharmacological activity, advances in nanocarrier-based delivery and balanced combination strategies have started to address critical challenges and limitations regarding solubility and bioavailability, while allowing precise therapeutic applications. In this review, we have discussed the plausible mechanisms, experimental evidence, and translational insights of eriodictyol as a systems-level modulator of cancer biology. We also outlined research priorities essential for progressing its clinical relevance as future perspectives.

## 1. Introduction

Natural products derived from plants have long been recognized as an important source of bioactive molecules investigated for their broad medical relevance across human diseases [1,2,3,4]. Owing to their chemical diversity and biological versatility, plant-derived compounds have been explored in multiple disease areas, including cancer, metabolic disorders, inflammatory conditions, and neurological diseases [5,6,7,8]. Recent advances in experimental biology and computational methodologies have further enabled the systematic investigation of natural compounds within complex disease-associated biological networks [4]. In parallel, continuous progress in biomedical research technologies—such as molecular profiling and spectroscopic approaches—has contributed to improved disease characterization and diagnostic exploration [9,10], while genomic analyses have enhanced understanding of disease-associated genetic alterations [11,12]. Moreover, growing insights into tumor heterogeneity, immune microenvironment interactions, and immunotherapy-related strategies have highlighted the importance of immune regulation in cancer research [13,14]. In addition to pharmacological agents, nutrition-derived biomolecules and naturally occurring peptides have also been investigated for their biological activity, reflecting the expanding scope of bioactive compounds examined in translational and preventive medicine [15]. Importantly, while these diverse studies illustrate the breadth of bioactive compounds and biomedical strategies under investigation, the therapeutic relevance and molecular mechanisms of individual natural compounds must be evaluated independently within defined disease contexts and supported by direct experimental evidence.

Among plant-derived secondary metabolites, flavonoids have emerged as a prominent class of compounds widely investigated for their structural diversity and broad biological activities across multiple disease-related pathways [16,17]. Their appeal is rooted in chemical diversity, safety, and capacity to contribute to multiple cellular processes that commonly help tumors to maintain their growth [18,19,20]. Plant-based compounds are increasingly understood not as isolated bioactives, but as modulators of interconnected cellular processes, including redox balance, apoptotic readiness, cell-cycle checkpoint integrity, and signaling adaptability [8,18,19,20]. This mode of action mirrors the systems-level complexity of cancer itself, where no single pathway operates in isolation [21]. Accordingly, recent work has moved away from cataloguing broad flavonoid pharmacology and toward identifying individual compounds that display reproducible, mechanism-driven effects across distinct tumor contexts. Particular attention has focused on flavonoids whose chemical features and biological behavior make them amenable to formulation engineering, targeted delivery, and eventual translational application, rather than remaining confined to descriptive in vitro activity [22].

Among the diverse flavonoid subclasses, eriodictyol is a naturally occurring flavanone that has garnered increasing research attention due to its well-defined chemical structure and reported biological activities in experimental studies [22,23,24]. Although it has long been discussed primarily in the context of antioxidant and anti-inflammatory activity outside oncology, its relevance to cancer biology has only come into focus more recently [22,23,24]. Accumulating evidence now shows that eriodictyol interferes with several molecular programs that malignant cells depend on for survival and adaptive fitness [22,23]. Rather than acting through a single pathway, its effects extend across key oncogenic and stress-response networks, including PI3K/Akt signaling [16], MAPK cascades, NF-κB-associated inflammatory circuits [17,18], inflammasome activity, and redox-regulated mechanisms linked to ferroptosis [3,13,14,15,16]. An additional and clinically relevant observation is its apparent preference for malignant cells, with multiple studies reporting greater sensitivity in tumor models compared with non-transformed counterparts [25,26,27]. This combination of mechanistic convergence and differential cytotoxicity distinguishes eriodictyol from more extensively studied flavonoids, whose anticancer effects often remain fragmented across isolated pathways without a unifying biological rationale [28,29].

Another critical development reshaping interest in eriodictyol is the emergence of nanomedicine-based delivery strategies [16,30]. As with many flavonoids, eriodictyol suffers from intrinsic limitations, poor aqueous solubility, rapid metabolism, and low systemic bioavailability [31,32]. Notably, a recent study in osteosarcoma reported that nano-co-delivery of eriodictyol with cisplatin potentiated ferroptotic cell death while mitigating off-target organ toxicity [33], an observation that elevates eriodictyol from a simple nutraceutical to a legitimate adjuvant candidate in combination therapy [34]. Such findings underscore the molecule’s translational potential and reinforce the necessity for an integrated, cancer-focused review that unites mechanistic, pharmacological, and delivery-science perspectives [33,34].

Despite the growing volume of experimental data, eriodictyol has remained largely peripheral in anticancer-focused reviews. Most existing discussions either subsume it within broad surveys of flavonoids or emphasize its antioxidant and anti-inflammatory roles, with oncologic relevance treated as incidental rather than central. Although interest in eriodictyol has expanded, the literature remains fragmented. Mechanistic insights are typically presented in isolation, making it difficult to identify shared pathways that operate across different cancer types. At the same time, practical constraints—most notably limited solubility and poor bioavailability—have received comparatively little attention, despite their obvious relevance for therapeutic translation. Evidence from in vivo models is still sparse, both in terms of antitumor efficacy and systemic safety. Moreover, eriodictyol’s potential alignment with emerging treatment paradigms, including ferroptosis-based strategies, immune modulation, and rational combination therapy, has yet to be evaluated in an integrated manner.

In response to these gaps, the present review brings together and critically examines the available evidence on eriodictyol as an emerging anticancer flavanone. Findings from cellular experiments, molecular analyses, and animal models are integrated to develop a unified perspective on how eriodictyol interferes with signaling programs that sustain malignant behavior. Particular emphasis is placed on its interactions with survival- and stress-adaptive pathways, as well as its ability to influence apoptotic commitment, migratory potential, and vulnerability to ferroptotic stress. In parallel, we examine advances in formulation and nano-enabled delivery approaches that seek to overcome pharmacokinetic barriers and enable therapeutic amplification. By situating eriodictyol within contemporary oncology concepts rather than treating it as an isolated natural product, this review aims to establish it as a mechanistically informative model for flavanone-based intervention and to define priorities for future translational investigation.

## 2. Chemical Structure and Pharmacokinetics of Eriodictyol

**Figure 1:**
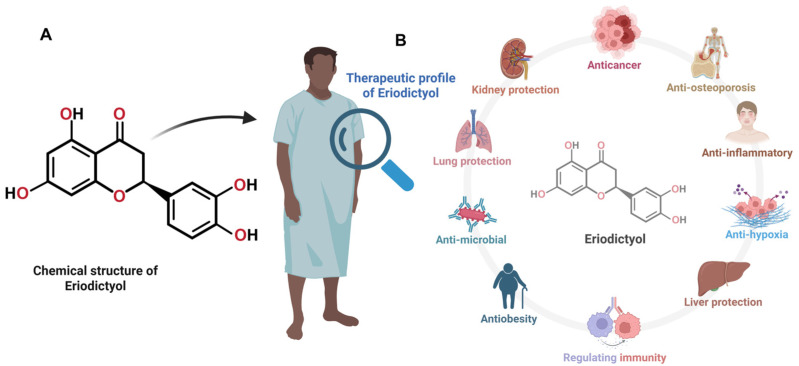
(**A**) Chemical structure of eriodictyol. (**B**) Therapeutic profile of eriodictyol. Created in BioRender. Mumtaz, S. (2026) https://BioRender.com/opmijwq (accessed on 10 February 2026).

Eriodictyol (5,7,3′,4′-tetrahydroxyflavanone) belongs to the flavanone subclass of flavonoids and is structurally characterized by a saturated C2–C3 bond, a chiral C2 center, and a catechol moiety on the B-ring [24,35] (Figure 1A). These features collectively influence its antioxidant behavior, radical-scavenging potential, metal-chelating ability, and its interactions with key signaling proteins. The presence of four hydroxyl groups confers high redox activity but simultaneously contributes to poor aqueous solubility and extensive phase II metabolism, constraints typical to many flavonoids [36]. Eriodictyol typically exists in free aglycone form or as glycosylated derivatives such as eriodictyol-7-O-glucoside and eriocitrin (eriodictyol-7-O-rutinoside), which are abundant in citrus fruits and herbal species, including *Eriodictyon californicum* and *Mentha* spp. [37,38].

From a biochemical perspective, the catechol (3′,4′-dihydroxy) arrangement on the B-ring is a defining determinant of eriodictyol’s reactivity. Catechol groups undergo rapid autoxidation, form o-quinones, modulate protein cysteine residues, and effectively participate in redox cycling, properties that partly explain the compound’s ability to activate apoptosis and regulate MAPK and PI3K/Akt pathways in cancer cells. Comparative structure–activity analyses among flavanones repeatedly point to the importance of B-ring substitution patterns in determining biological potency. Flavanones bearing a catechol group on the B ring repeatedly show superior performance relative to mono-hydroxylated counterparts in assays probing anticancer activity, redox regulation, and inflammatory control [39,40]. This advantage is not incidental. The catechol motif enhances electron-donating capacity and promotes interaction with redox-sensitive signaling elements as well as metal-dependent enzymatic systems, providing a plausible mechanistic basis for its biological potency.

Direct pharmacokinetic characterization of eriodictyol remains limited, yet available studies reveal a consistent in vivo pattern (Figure 1B). After oral administration in rodent models, the compound is absorbed quickly but eliminated just as rapidly, exhibiting a systemic half-life of under two hours [36,41]. Such transient exposure reflects the combined effects of poor aqueous solubility and extensive first-pass metabolism, traits typical of flavanone aglycones. As a result, circulating levels remain relatively low, highlighting the challenge of sustaining therapeutically meaningful concentrations through standard oral dosing. Distribution analyses further indicate preferential accumulation in metabolically active tissues, helping to contextualize eriodictyol’s in vivo behavior [24,42].

Viewed from a translational standpoint, eriodictyol occupies an unusual position within the flavanone class. Its catechol-rich benzopyran scaffold supports strong redox activity and engagement of cancer-relevant signaling pathways, yet this biological potential is counterbalanced by rapid metabolic turnover. The resulting disconnect between molecular capability and pharmacological exposure underscores a broader implication: progress is more likely to come from advances in delivery and formulation strategies than from structural modification alone.

## 3. Eriodictyol and Cancer Cell Fate Control

### 3.1. Eriodictyol as a Regulator of the Cell Cycle

**Figure 2:**
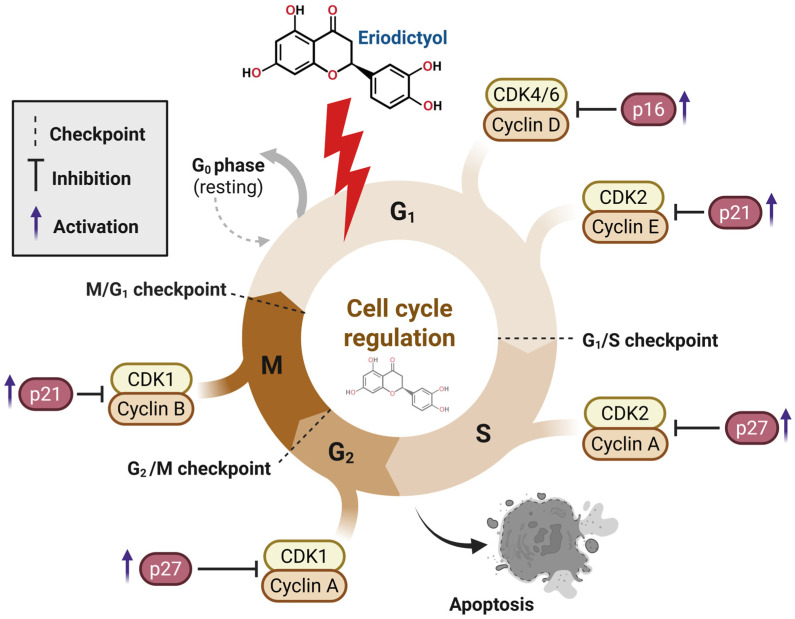
Conceptual overview of eriodictyol-induced disruption of cell-cycle control and proliferative signaling in cancer cells. Eriodictyol interferes with cancer cell proliferation by imposing multi-level constraints on cell-cycle progression rather than acting through a single checkpoint blockade. As illustrated, eriodictyol attenuates the activity of cyclin–CDK complexes required for orderly transition through G1, S, G2, and M phases, while concurrently reinforcing endogenous CDK inhibitory programs. Suppression of early-phase cyclin D–CDK4/6 and cyclin E–CDK2 activity limits cell-cycle entry and S-phase commitment, whereas inhibition of cyclin A- and cyclin B-associated complexes restricts replication completion and mitotic initiation. These checkpoint constraints are mechanistically coupled to reduced PI3K/Akt/mTOR signaling, thereby linking growth-factor attenuation to restored checkpoint fidelity. Prolonged arrest under these conditions destabilizes mitochondrial homeostasis and lowers the apoptotic threshold, ultimately shifting cellular fate away from division and toward programmed cell death. Created in BioRender. Mumtaz, S. (2026) https://BioRender.com/opmijwq.

Eriodictyol is now being viewed less as a simple growth-inhibitory flavanone and more as a compound that interferes with the decision-making machinery underlying cancer cell division [27,43]. Rather than imposing non-selective cytotoxic stress, the available data suggest that its primary action occurs upstream of mitotic collapse, at the level of cell-cycle licensing and checkpoint governance. This distinction matters because sustained tumor expansion depends not only on rapid proliferation but on the ability of malignant cells to override checkpoint controls and continue cycling despite genomic instability or metabolic strain. Across a range of cancer models, eriodictyol has repeatedly been shown to restrain progression at key transition points, most notably the G1/S and G2/M interfaces [44,45]. These checkpoints function as integrative hubs, where signals related to growth cues, DNA integrity, and cellular fitness are weighed before cells commit to replication or mitosis [44,45]. In cancer, these safeguards are often weakened, allowing division to proceed under conditions that would normally trigger arrest or elimination. Eriodictyol appears to reverse this permissive state by re-imposing checkpoint pressure, thereby curbing unregulated cycle progression and increasing susceptibility to cell death. At a mechanistic level, this effect reflects coordinated disruption of cyclin–CDK signaling and its upstream regulatory inputs [27,46,47,48]. Experimental studies describe reduced activity or expression of cyclins and CDKs responsible for S-phase entry and mitotic initiation, alongside reinforcement of endogenous inhibitory controls. Notably, these cell-cycle effects are rarely isolated events. They frequently coincide with dampening of survival pathways—particularly those converging on PI3K/Akt/mTOR—indicating that eriodictyol couples growth arrest with erosion of pro-survival signaling capacity. As outlined schematically in Figure 2, this layered interference diminishes proliferative momentum and steers cells toward apoptotic resolution rather than transient arrest [49].

#### 3.1.1. Rewiring Cyclin–CDK Control Nodes by Eriodictyol

Sustained proliferation in cancer relies on chronic activation of cyclin–CDK assemblies that, under physiological conditions, act as tightly timed regulators of cell division [22,48,49]. Instead of engaging a single cyclin or kinase node, eriodictyol appears to place distributed pressure on this control system, attenuating several cyclin-dependent transitions while simultaneously strengthening intrinsic inhibitory constraints [22,48,49]. This broader mode of interference sets eriodictyol apart from conventional CDK inhibitors, which often act with narrow specificity and are prone to rapid compensatory bypass. Across multiple tumor models, eriodictyol has been reported to reduce the expression or functional activity of cyclins required for orderly cell-cycle progression, from early commitment events through preparation for mitosis. Diminished activity of cyclin D- and cyclin E-associated kinases compromises initial entry into the cycle, while suppression of later-phase cyclins limits completion of replication and readiness for mitotic transition. These effects coincide with increased levels of endogenous CDK inhibitors, including p16, p21, and p27, which together reinforce checkpoint stringency and restrain oncogenic reactivation of CDK signaling. The net outcome is a gradual contraction of permissive cycling states rather than an abrupt, nonspecific cytotoxic halt.

At the G1 restriction point, eriodictyol restricts cyclin D–CDK4/6 signaling and favors accumulation of p16, maintaining retinoblastoma protein in a hypophosphorylated, growth-restrictive configuration. This delays progression into S phase and limits inappropriate replication initiation. Additional control is imposed at the G1/S boundary through inhibition of cyclin E–CDK2 complexes, reinforced by elevated p21 expression that further restrains DNA synthesis [48,50]. As cells advance, eriodictyol continues to erode proliferative drive by weakening cyclin A-dependent complexes and enhancing p27-mediated inhibition, ultimately converging on suppression of cyclin B–CDK1 activity at the G2/M interface. This late-stage blockade prevents mitotic entry and biases cellular fate toward apoptotic resolution rather than division (Figure 2) [23]. Importantly, these checkpoint effects are embedded within broader signaling changes. In several systems, eriodictyol attenuates PI3K/Akt/mTOR activity, a pathway that normally sustains cyclin production, CDK activation, and checkpoint escape. Suppression of this axis destabilizes cyclin expression while favoring CDK inhibitor accumulation, mechanistically linking growth-factor signaling restraint to restoration of checkpoint fidelity. Through this integrated reprogramming, eriodictyol effectively turns proliferative advantage into a liability for cancer cells.

#### 3.1.2. Checkpoint Enforcement as a Trigger for Apoptotic Commitment

The G1/S and G2/M checkpoints operate as critical control points where cues promoting proliferation are balanced against DNA integrity and cellular fitness. In malignant cells, these safeguards are frequently compromised, permitting continued division under conditions that would ordinarily trigger arrest or elimination [22,27,51]. Eriodictyol appears to take advantage of this weakened checkpoint architecture by restoring regulatory pressure, although the dominant site of arrest varies with tumor type and oncogenic context. Evidence from multiple cancer models shows that eriodictyol can restrain progression at both early and late stages of the cell cycle [22,27,51]. In some systems, inhibition of cyclin D- and cyclin E-dependent kinase activity limits commitment to S phase and blocks replication onset. In others, stronger effects are observed at the G2/M transition, where suppression of cyclin B–CDK1 activity prevents entry into mitosis [22]. These patterns are not mutually exclusive and may coexist within the same cellular background, reflecting differential checkpoint sensitivity rather than distinct mechanisms of action [26].

Importantly, cell-cycle arrest induced by eriodictyol does not persist as a neutral, reversible pause. Sustained checkpoint enforcement progressively destabilizes mitochondrial homeostasis, shifts BCL-2 family signaling toward a pro-apoptotic balance, and facilitates caspase activation. This evolution from arrest to execution distinguishes eriodictyol from agents that transiently suppress proliferation without resolving cell fate. Concurrent attenuation of PI3K/Akt/mTOR signaling further erodes survival capacity, limiting the ability of arrested cells to recover or adapt. As a consequence, checkpoint engagement and apoptotic commitment become functionally intertwined rather than sequential or independent events [46]. By coupling cell-cycle restraint with programmed cell death, eriodictyol transforms proliferative dependence into a vulnerability, enforcing irreversible growth suppression rather than temporary inhibition.

### 3.2. Regulation of Intrinsic and Extrinsic Apoptotic Pathways by Eriodictyol

Malignant cells frequently avoid apoptotic elimination by stabilizing mitochondrial integrity and dampening death-receptor responsiveness, effectively elevating the threshold required to initiate programmed cell death [52]. Evidence suggests that eriodictyol undermines this adaptive advantage by activating apoptotic signaling through more than one entry point, limiting the ability of cancer cells to rely on a single escape route. At the mitochondrial level, eriodictyol disrupts homeostatic control by rebalancing BCL-2 family proteins, promoting membrane permeabilization, release of cytochrome c, and activation of downstream caspases [52]. This mitochondrial priming is commonly accompanied by suppression of survival pathways that would otherwise buffer cells against apoptotic commitment [53,54]. Concurrently, eriodictyol enhances responsiveness to death receptor-initiated signaling, strengthening caspase activation and broadening the spectrum of extracellular cues capable of driving cell death. The integration of mitochondrial and receptor-dependent inputs at shared executioner caspases results in efficient apoptotic completion rather than partial or abortive signaling. Importantly, this mode of action reflects restoration of apoptotic capacity rather than indiscriminate cytotoxic stress [55,56]. Such dual-pathway engagement is especially pertinent in treatment-refractory tumors, where dependence on a single apoptotic mechanism is often compromised, and redundancy becomes essential for effective elimination.

#### 3.2.1. Activation of the Intrinsic (Mitochondrial) Apoptotic Pathway

**Figure 3:**
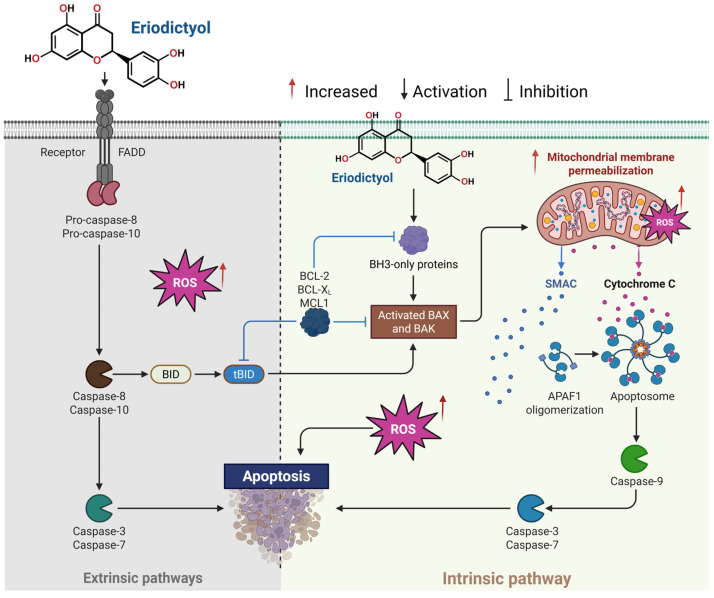
Mechanistic overview of eriodictyol-induced intrinsic and extrinsic apoptotic pathways in cancer cells. Eriodictyol activates both extrinsic and intrinsic apoptotic pathways. In the extrinsic pathway, eriodictyol promotes death receptor-mediated recruitment of FADD and activation of caspase-8/10, leading to direct executioner caspase activation and cleavage of BID to truncated BID (tBID). tBID translocates to mitochondria, where eriodictyol simultaneously modulates BCL-2 family proteins by suppressing BCL-2, BCL-XL, and MCL-1 and activating BAX/BAK, resulting in mitochondrial outer membrane permeabilization. This process releases cytochrome c and Second Mitochondria-derived Activator of Caspases (SMAC), triggering APAF1 oligomerization, apoptosome formation, caspase-9 activation, and downstream caspase-3/7 execution. Controlled ROS elevation further amplifies mitochondrial dysfunction and apoptotic commitment. Crosstalk between extrinsic and intrinsic pathways ensures efficient apoptosis and suppression of cancer cell survival. Created in BioRender. Mumtaz, S. (2026) https://BioRender.com/opmijwq.

Intrinsic apoptosis is typically initiated when malignant cells are exposed to persistent internal stress, such as oxidative disequilibrium, unresolved DNA damage, or metabolic strain that exceeds adaptive capacity [55,56,57,58]. Under these conditions, mitochondrial homeostasis deteriorates, enabling the release of pro-apoptotic mediators that lock the cell into an irreversible death trajectory. Eriodictyol capitalizes on this intrinsic vulnerability by directly perturbing mitochondrial stability and increasing membrane permeability, events that culminate in cytochrome c efflux, as illustrated in Figure 3. At the signaling level, this mitochondrial destabilization coincides with a decisive rebalancing of BCL-2 family regulators. Pro-apoptotic factors such as BAX and BAK are upregulated, while anti-apoptotic proteins, including BCL-2, BCL-XL, and MCL-1, are concurrently suppressed, collectively lowering the threshold for mitochondrial outer membrane permeabilization [27]. This molecular shift enables efficient apoptosome assembly, thereby facilitating robust activation of the caspase cascade [59]. Following cytochrome c release, eriodictyol promotes oligomerization of apoptotic protease-activating factor-1 (APAF1) and subsequent activation of caspase-9, which in turn drives executioner caspases-3 and -7 [25,27,55,60]. The resulting cascade produces classical apoptotic hallmarks, including chromatin fragmentation, cytoskeletal collapse, and orderly cellular dismantling, confirming engagement of a fully competent mitochondrial death program. Importantly, eriodictyol-induced intrinsic apoptosis is closely linked to ROS modulation, where controlled ROS elevation contributes to mitochondrial dysfunction and apoptotic commitment in cancer cells, in contrast to normal cells, where antioxidant defenses may remain intact [51].

#### 3.2.2. Engagement of the Extrinsic Apoptotic Pathway and Pathway Crosstalk

In addition to mitochondrial apoptosis, eriodictyol can activate the extrinsic apoptotic pathway through death receptor signaling at the plasma membrane [48]. The extrinsic pathway is initiated by ligand-mediated activation of death receptors, leading to recruitment of adaptor proteins such as Fas-Associated protein with Death Domain (FADD) and subsequent activation of initiator caspases-8 and -10 [55]. Evidence indicates that eriodictyol enhances death receptor-mediated apoptotic signaling by facilitating caspase-8 activation, which directly activates executioner caspases and indirectly amplifies mitochondrial apoptosis through BID cleavage to truncated BID (tBID), thereby linking extrinsic and intrinsic pathways (Figure 3). This bidirectional crosstalk is particularly relevant in cancer cells exhibiting resistance to single-pathway apoptotic triggers. By activating death receptor-associated caspase-8 signaling alongside BAX/BAK-driven mitochondrial disruption, eriodictyol effectively builds redundancy into the apoptotic response [61]. This dual engagement limits the capacity of cancer cells to evade death by disabling a single pathway. Signals from both routes ultimately converge on executioner caspases, particularly caspase-3 and caspase-7, ensuring that apoptotic commitment proceeds efficiently once initiated [61]. Through this coordinated activation, eriodictyol narrows the survival margin of tumor cells and constrains adaptive resistance mechanisms.

#### 3.2.3. Redox-Dependent Apoptotic Sensitization and Pathway Selectivity

Reactive oxygen species (ROS) occupy a paradoxical position in cancer biology, supporting signal transduction at controlled levels while becoming lethal when oxidative balance is pushed beyond tolerance [62]. Eriodictyol appears to exploit this imbalance by reshaping intracellular redox dynamics in a context-dependent manner [63,64]. In cancer cells, where basal oxidative stress is already elevated, eriodictyol further perturbs redox homeostasis, promoting mitochondrial depolarization, caspase activation, and apoptotic progression [63,64]. In contrast, non-malignant cells exhibit greater redox buffering capacity, which may explain the relative selectivity observed across experimental models [65]. This redox-driven vulnerability is closely coupled to suppression of oncogenic survival signaling. Eriodictyol-mediated attenuation of PI3K/Akt/mTOR activity weakens anti-apoptotic defenses, destabilizes BCL-2 family proteins, and lowers the threshold required for apoptotic commitment [25]. Rather than acting through ROS generation alone, eriodictyol integrates oxidative stress with signaling inhibition, creating conditions in which mitochondrial dysfunction and caspase activation become difficult to reverse.

Taken together, eriodictyol functions as a multi-layered apoptotic sensitizer, coordinating redox modulation with mitochondrial and receptor-mediated death signaling while simultaneously suppressing survival pathways [64,66]. This convergence provides a mechanistic basis for its activity in apoptosis-resistant tumor settings and supports its rational inclusion in combination strategies aimed at overcoming entrenched survival signaling.

### 3.3. Inhibition of Angiogenesis and Metastatic Progression by Eriodictyol

Angiogenesis and metastasis are central to cancer lethality, as they enable sustained tumor growth and facilitate dissemination beyond the primary site. Accumulating evidence shows that eriodictyol can effectively interfere with both processes by targeting vascular signaling, extracellular matrix remodeling, and epithelial–mesenchymal transition (EMT), thereby inhibiting tumor progression at various stages [67,68].

**Figure 4:**
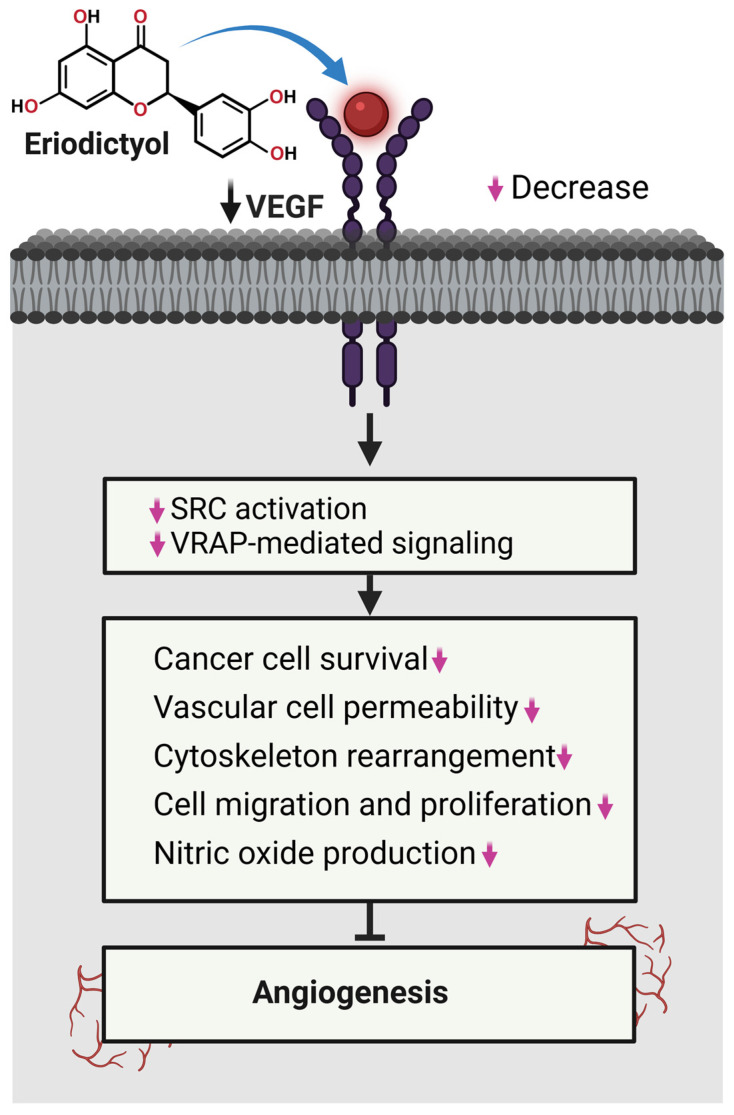
The effects of eriodictyol on VEGF signaling and angiogenesis in cancer cells. Eriodictyol inhibits VEGF signaling, resulting in the downregulation of SRC, Sck, and VEGF Receptor-Associated Protein (VRAP) activities. This suppression reduces cell survival, vascular permeability, cytoskeletal changes, migration, proliferation, and nitric oxide production, ultimately blocking angiogenesis. Created in BioRender. Mumtaz, S. (2026) https://BioRender.com/opmijwq.

Rather than acting at a single step, eriodictyol constrains multiple components of the angiogenic–metastatic axis, limiting tumor expansion and migratory capacity, as outlined in Figure 4. At the vascular level, eriodictyol suppresses angiogenic drive by attenuating signaling pathways that converge on vascular endothelial growth factor (VEGF) expression. Inhibition of PI3K/Akt-, STAT3-, and ERK-associated transcriptional activity reduces endothelial activation and neovascular support within the tumor microenvironment [69,70]. In parallel, eriodictyol impairs extracellular matrix remodeling by downregulating key matrix metalloproteinases (MMPs), particularly MMP-2 and MMP-9, thereby restricting basement membrane degradation and invasive progression [71,72]. These effects are frequently accompanied by a shift toward anti-angiogenic and anti-invasive signaling states, further stabilizing tissue barriers against tumor dissemination [73,74,75,76]. Through this coordinated suppression of vascular expansion and matrix-driven invasion, eriodictyol targets core biological requirements for metastatic competence, reinforcing its role as a multi-level modulator of tumor progression rather than a pathway-restricted inhibitor. Through these effects, eriodictyol limits both vessel formation and extracellular matrix remodeling required for metastatic escape.

Beyond its effects on tumor vascularization, eriodictyol also constrains metastatic potential by limiting epithelial–mesenchymal plasticity, a prerequisite for efficient cancer cell dissemination [76]. Across multiple models, eriodictyol reinforces epithelial identity by restoring E-cadherin expression while suppressing mesenchymal markers associated with motility and invasion. This phenotypic stabilization reduces the capacity of tumor cells to detach, migrate, and adapt to hostile microenvironments [76]. Mechanistically, suppression of EMT by eriodictyol is closely aligned with attenuation of signaling pathways that coordinate survival, cytoskeletal remodeling, and migratory behavior, including PI3K/Akt-, p38 MAPK-, and STAT3-dependent networks [75]. In parallel, eriodictyol disrupts adhesion-associated signaling and cytoskeletal dynamics, further diminishing invasive competence. Rather than selectively targeting a single metastatic effector, eriodictyol undermines the integrated signaling architecture that supports EMT-driven dissemination [75]. Through this coordinated restraint of plasticity and motility, eriodictyol emerges as a functionally relevant suppressor of metastatic progression and a plausible adjunct for limiting tumor spread in advanced disease settings.

### 3.4. Redox and Inflammatory Signaling Modulation by Eriodictyol in Cancer

Oxidative stress and chronic inflammation are closely linked factors that drive tumor initiation, progression, and resistance to therapy [23,77]. Cancer cells frequently exploit dysregulated redox signaling and inflammatory pathways to sustain proliferation, evade apoptosis, and promote angiogenesis and metastasis [23]. Eriodictyol appears to act in a highly context-dependent manner, limiting tumor progression not by functioning as a simple antioxidant, but by recalibrating redox balance and dampening inflammatory signaling pathways that otherwise support malignant growth.

#### 3.4.1. Redox Homeostasis as a Therapeutic Vulnerability

Eriodictyol modulates intracellular redox balance through a combination of direct radical buffering and regulation of endogenous antioxidant defenses. Beyond its intrinsic electron-donating capacity, eriodictyol influences redox control by engaging transcriptional programs that govern cellular antioxidant responses, most notably those linked to Nrf2 signaling [78]. Activation of this pathway enhances the expression of enzymes involved in reactive species detoxification and mitochondrial stabilization, thereby preventing pathological amplification of oxidative stress [79]. Importantly, this activity does not translate into uniform antioxidant suppression. In non-malignant or inflamed tissues, redox stabilization confers cytoprotection, whereas in cancer cells, where oxidative pressure is already elevated, eriodictyol disrupts adaptive redox tolerance. This imbalance lowers the threshold for stress-induced cell death, sensitizing malignant cells to apoptotic or ferroptotic pathways [80]. Such context selectivity underscores eriodictyol’s function as a redox regulator rather than a passive scavenger.

#### 3.4.2. Attenuation of Pro-Tumorigenic Inflammatory Signaling

Sustained inflammatory signaling supports tumor progression by reinforcing survival pathways, promoting angiogenesis, and shaping an immunosuppressive microenvironment [81]. Eriodictyol interferes with these processes by dampening key inflammatory signaling axes, including NF-κB-driven transcription and COX-2-dependent prostaglandin synthesis. Suppression of these pathways limits the production of pro-inflammatory cytokines and disrupts feedback loops that otherwise sustain tumor-supportive inflammation [81]. In parallel, eriodictyol reduces excessive reactive oxygen species generation by modulating ROS-producing enzymes, further constraining inflammation-linked signaling amplification within the tumor microenvironment (TME) [82]. Taken together, eriodictyol operates at the intersection of redox regulation and inflammatory control [83]. Rather than indiscriminately silencing oxidative or immune signaling, it selectively recalibrates stress-responsive pathways in a manner that undermines malignant adaptation while preserving physiological balance [83]. This integrative mode of action reinforces its potential value as an anticancer sensitizer and adjunct therapeutic agent.

### 3.5. Modulation of MAPK Signaling by Eriodictyol

Mitogen-activated protein kinase (MAPK) signaling functions as a central integration platform through which oncogenic growth cues, stress signals, inflammatory inputs, and fate-determining decisions are coordinated [84]. Persistent dysregulation of MAPK branches, most notably Extracellular Signal-Regulated Kinase (ERK), c-Jun N-terminal Kinase (JNK), and p38, is a recurring feature of malignant progression, underpinning uncontrolled proliferation, resistance to cell death, invasive behavior, and adaptive therapy escape [85]. Within this framework, accumulating evidence suggests that eriodictyol does not uniformly suppress MAPK activity but instead reshapes pathway output in a context-dependent manner that favors growth restraint and apoptotic commitment [85,86]. In highly proliferative tumor settings, eriodictyol dampens aberrant ERK-driven signaling that sustains mitogenic transcriptional programs and supports migratory capacity [87]. Attenuation of ERK activity is accompanied by reduced expression of downstream regulators associated with cell-cycle progression and motility, reinforcing eriodictyol’s antiproliferative and anti-metastatic profile [88,89]. Notably, ERK suppression rarely occurs in isolation; it frequently coincides with inhibition of convergent survival pathways, including PI3K/Akt/mTOR signaling [88,89]. This coordinated downregulation suggests that eriodictyol constrains oncogenic network redundancy rather than targeting a single signaling axis.

In contrast to its inhibitory effects on ERK-mediated survival, eriodictyol selectively enhances stress-responsive MAPK branches, particularly JNK and p38, within malignant cells [88,89]. Activation of these kinases promotes transcriptional programs associated with cellular stress resolution and apoptosis, including phosphorylation of c-Jun and p53, induction of pro-apoptotic mediators such as BAX and PUMA, and suppression of anti-apoptotic BCL-2 family members [51]. Engagement of JNK- and p38-dependent signaling contributes to mitochondrial destabilization, caspase activation, and irreversible apoptotic progression [51]. Importantly, this pro-death MAPK activation appears preferentially amplified in cancer cells, likely reflecting their heightened sensitivity to redox imbalance and metabolic stress [90]. Beyond direct control of cell fate, eriodictyol-mediated MAPK remodeling intersects with inflammatory regulation [66,91]. By limiting MAPK-dependent activation of NF-κB, eriodictyol reduces pro-inflammatory cytokine output and weakens inflammation-driven support within the TME [86,92]. This coordinated suppression of MAPK and NF-κB signaling further constrains angiogenesis, invasion, and establishment of metastatic niches.

Taken together, these findings identify eriodictyol as a selective reprogrammer of MAPK signaling, capable of restraining proliferative ERK outputs while amplifying stress-induced JNK/p38 apoptotic responses. Such balanced modulation disrupts oncogenic signaling plasticity and supports the positioning of eriodictyol as a multi-pathway anticancer sensitizer rather than a single-node inhibitor.

## 4. Therapeutic Applications of Eriodictyol in Precision Oncology

A growing number of preclinical in vitro studies show eriodictyol has cytotoxic, anti-proliferative, anti-migratory/invasive, and pro-apoptotic effects on various human cancer cell lines. While the spectrum is broad, including lung carcinoma, glioma, pancreatic cancer, hepatocellular carcinoma, and colorectal cancer, among others, consistent mechanistic themes emerge. In many cases, eriodictyol suppresses oncogenic signaling (e.g., PI3K/Akt and NF-κB), triggers mitochondrial or intrinsic apoptosis, interferes with cell-cycle progression, and inhibits metastatic phenotypes (migration, invasion, colony formation) [93].

**Figure 5:**
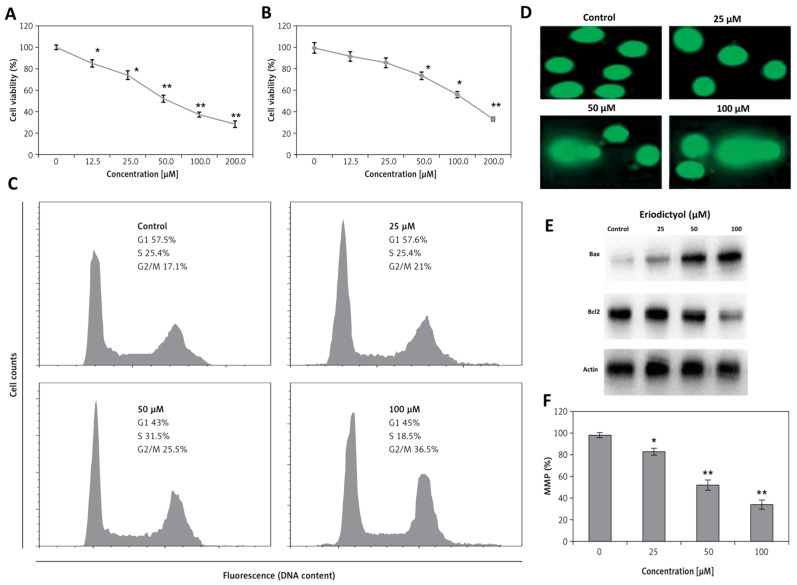
Antitumor activity of eriodictyol in A549 NSCLC cells. (**A**) Eriodictyol reduces A549 cell viability in a concentration-dependent manner, with an estimated IC_50_ of approximately 50 μM. (**B**) In contrast, non-malignant FR2 lung epithelial cells display substantially lower sensitivity, indicating a degree of tumor selectivity. (**C**) Cell-cycle analysis reveals a progressive enrichment of eriodictyol-treated A549 cells at the G2/M phase, consistent with activation of a DNA damage-associated checkpoint. (**D**) Representative comet assay images show dose-dependent DNA fragmentation, supporting the induction of genotoxic stress. (**E**) Immunoblotting demonstrates a shift toward a pro-apoptotic balance, characterized by increased Bax expression and concomitant suppression of Bcl-2. (**F**) Quantification of mitochondrial membrane potential (MMP) demonstrates dose-dependent mitochondrial depolarization. Adopted from [27]. (Statistical significance is indicated as * *p* < 0.05 and ** *p* < 0.01).

One of the earliest mechanistic studies highlighting eriodictyol’s anticancer relevance was conducted in human lung adenocarcinoma. Zhang et al. reported that, in A549 lung carcinoma cells, eriodictyol orchestrates a tightly connected cascade of antiproliferative and pro-apoptotic events that together define a robust cytotoxic phenotype (Figure 5) [27]. Eriodictyol produces a clear, concentration-dependent loss of viability in A549 lung cancer cells (Figure 5A), while exerting comparatively modest effects on non-malignant FR2 epithelial cells (Figure 5B) [27]. This differential sensitivity is notable, as many naturally derived compounds fail to discriminate effectively between malignant and normal epithelial populations [27]. The early cellular response to eriodictyol exposure is characterized by the emergence of genotoxic stress, reflected by progressive DNA fragmentation detected by comet assay (Figure 5C,D). This damage coincides with a marked accumulation of cells at the G2/M boundary, suggesting activation of a checkpoint-mediated attempt to halt division and preserve genomic integrity. However, this response appears transient [27]. The subsequent loss of mitochondrial membrane potential indicates that repair mechanisms are insufficient, shifting cellular fate from arrest toward irreversible apoptotic promise [60]. Consistent with this transition, eriodictyol treatment drives a pronounced redistribution of BCL-2 family signaling, favoring a pro-apoptotic state through elevation of Bax relative to Bcl-2 (Figure 5E,F) [27]. These changes position mitochondrial destabilization as a decisive event in eriodictyol-induced cell death. Rather than acting through a single lesion, eriodictyol coordinates nuclear stress signaling, checkpoint enforcement, and mitochondrial execution into a tightly linked cascade [94,95,96]. Such multi-tiered engagement is particularly relevant in non-small-cell lung cancer, where robust survival circuitry—most notably PI3K/Akt/mTOR signaling—often blunts isolated therapeutic insults [27]. By simultaneously compromising genomic stability and mitochondrial homeostasis, eriodictyol appears to circumvent this buffering capacity and drive apoptosis through routes that are difficult for tumor cells to bypass.

From a translational standpoint, these findings elevate eriodictyol beyond a simple cytotoxic flavanone. Compounds that impose G2/M arrest while destabilizing mitochondrial function frequently cooperate with DNA-damaging agents, raising the possibility that eriodictyol could sensitize non-small-cell lung cancer (NSCLC) to platinum-based chemotherapy [27]. The relative sparing of non-malignant epithelial cells further suggests a potentially favorable therapeutic margin. Future work should determine whether eriodictyol interferes with PI3K/Akt-mediated resistance programs, enhances cisplatin responsiveness, and maintains efficacy across genetically diverse NSCLC subtypes, including KRAS- and EGFR-driven tumors.

In this context, eriodictyol displayed a clear preference for malignant cells, reducing A549 viability with an IC_50_ of approximately 50 µM, whereas non-transformed FR2 epithelial cells were considerably less affected, with inhibitory concentrations close to 95 µM. Exposure to eriodictyol triggered a pronounced mitochondrial stress response, evidenced by collapse of membrane potential, increased Annexin-V positivity, and a decisive shift in the Bax/Bcl-2 balance toward apoptosis. These mitochondrial alterations coincided with cell-cycle blockade at the G2/M transition and progression toward apoptotic cell death. Notably, these downstream events were paralleled by strong attenuation of PI3K/Akt/mTOR signaling, indicating that eriodictyol disrupts oncogenic growth cues upstream of mitochondrial commitment rather than acting solely at the execution phase of apoptosis [27].

**Figure 6:**
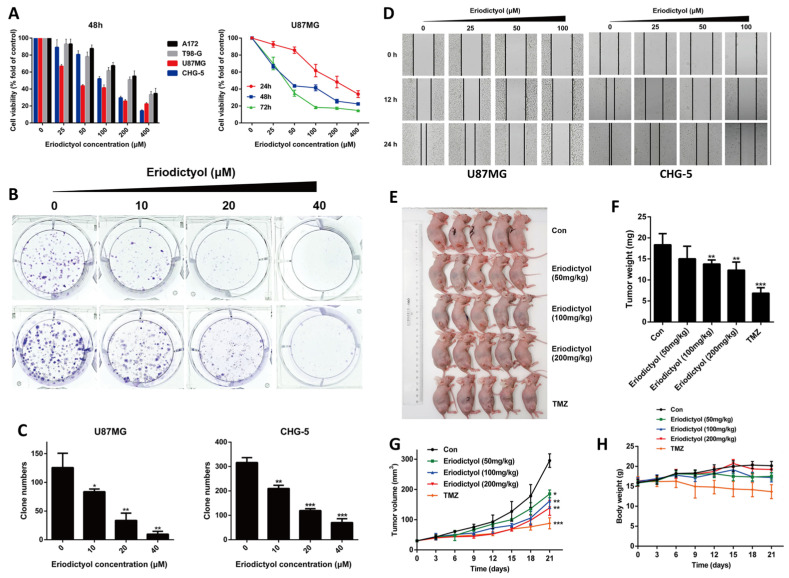
Eriodictyol suppresses glioma growth, clonogenicity, invasion, and tumor progression in vitro and in vivo. Eriodictyol reduces glioma cell viability and long-term clonogenic survival across multiple glioma models (**A**–**C**) and markedly attenuates migratory capacity (**D**). In vivo, eriodictyol treatment suppresses tumor growth and tumor burden in xenograft models (**E**–**G**) without inducing significant systemic toxicity, as reflected by stable body weight profiles (**H**). Together, these data support eriodictyol as a multi-target inhibitor of glioma progression with translational relevance. Adopted from [25]. (Differences between groups are denoted by * *p* < 0.05, ** *p* < 0.01, and *** *p* < 0.001).

Li et al. presented a substantive body of evidence indicating that eriodictyol acts as a multi-dimensional inhibitor of glioma aggressiveness rather than a simple antiproliferative agent [25]. Across several glioma models, eriodictyol consistently impaired cellular fitness, suppressing short-term viability as well as long-term clonogenic capacity (Figure 6A–C), which suggests engagement of core survival dependencies shared across glioma subtypes. Of particular relevance to glioblastoma biology, eriodictyol markedly restrained migratory behavior (Figure 6D), a feature intimately linked to diffuse brain infiltration and therapeutic failure in clinical settings. The translational weight of this study is strengthened by its in vivo component, where eriodictyol produced a clear reduction in tumor burden without evidence of systemic toxicity (Figure 6E–H) [25]. Such concurrent suppression of proliferation, invasion, and tumor growth in animal models remains uncommon among dietary flavonoids, positioning eriodictyol as a comparatively advanced candidate within this chemical class. Mechanistic analyses linked these phenotypic outcomes to coordinated disruption of PI3K/Akt/NF-κB signaling alongside activation of mitochondrial apoptotic pathways, underscoring eriodictyol’s ability to dismantle parallel survival circuits that typically confer robustness to glioma cells [25].

Conceptually, this work is important because it reframes eriodictyol from a generic antioxidant into a signal-interfering molecule with tangible oncologic relevance. The combined inhibition of clonogenic persistence and invasive capacity implies potential utility not only in reducing tumor mass but also in constraining recurrence and infiltrative spread. At the same time, the modest potency relative to standard agents such as temozolomide highlights a clear development path forward, emphasizing rational combination strategies, nanoformulation approaches, and optimization for blood–brain barrier penetration to unlock its full therapeutic potential [25]. Future investigations should prioritize orthotopic glioma models, mechanistic integration with DNA damage responses, and evaluation of synergy with standard-of-care chemotherapeutics to exploit eriodictyol’s therapeutic promise fully.

**Figure 7:**
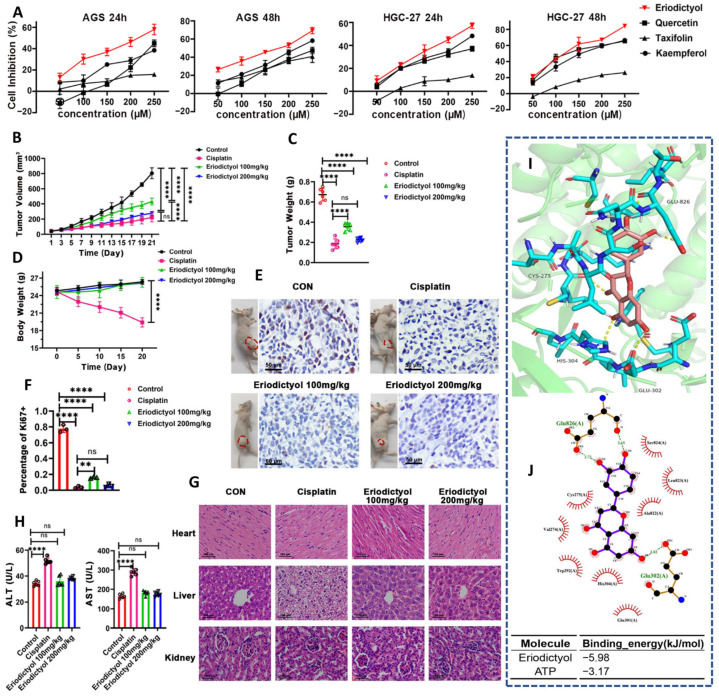
Eriodictyol exhibits superior anticancer efficacy and PI3K/AKT pathway targeting in gastric cancer models. Eriodictyol displays stronger growth-inhibitory activity than structurally related flavonoids in gastric cancer cells (**A**) and suppresses xenograft tumor growth and burden in vivo in a dose-dependent manner. (**B**,**C**) Tumor volume and weight, (**D**) body-weight loss. (**E**,**F**) Reduced tumor proliferative capacity is confirmed by Ki-67 immunostaining (Scar bar: 50 μm). (**G**,**H**) Histological and biochemical assessments demonstrate minimal toxicity to major organs (Scar bar: 100 μm). (**I**,**J**) Systems pharmacology and molecular docking analyses identify PI3K as a central molecular target. Together, these data establish eriodictyol as a pathway-directed flavonoid with a favorable efficacy–safety balance in gastric cancer. Adopted from [26]. (Differences between groups are denoted by ‘ns’: non-significant, ** *p* < 0.01, and **** *p* < 0.0001).

In gastric cancer models, eriodictyol was identified as the most potent anticancer constituent among major flavonoids derived from Polygoni orientalis Fructus (Figure 7A–J), consistently outperforming quercetin, taxifolin, and kaempferol in suppressing tumor cell growth across AGS and HGC-27 cells (Figure 7A) [26]. This comparative advantage is noteworthy because it situates eriodictyol not simply as another bioactive flavonoid, but as a molecule whose structural features translate into measurable mechanistic efficiency against gastric cancer. The superior antiproliferative activity observed in vitro was recapitulated in vivo, where eriodictyol produced a sustained, dose-responsive reduction in xenograft tumor growth (Figure 7B) [26]. Strikingly, the reduction in tumor mass approached that achieved with cisplatin (Figure 7C), yet occurred in the absence of the systemic toxicity typically associated with platinum-based therapy (Figure 7D) [26].

Importantly, eriodictyol-treated tumors exhibited a pronounced reduction in Ki-67 staining (Figure 7E,F), indicating effective suppression of tumor cell cycling in vivo [26]. From a translational standpoint, the safety profile of eriodictyol further strengthens its candidacy. Histological evaluation revealed preserved architecture in key organs, including liver, kidney, and heart (Figure 7G) [26], accompanied by stable serum transaminase levels (Figure 7H). This contrasts sharply with the organ stress associated with cisplatin exposure and underscores a favorable therapeutic index. At the mechanistic level, tumor suppression was linked to focused interference with PI3K/AKT signaling. Network pharmacology and protein–protein interaction analyses converged on PI3K as a dominant regulatory node influenced by eriodictyol (Figure 7I) [26]. This prediction was supported by molecular docking simulations demonstrating stable occupancy of the PI3K catalytic domain by eriodictyol with favorable binding energetics (Figure 7J). 

Conceptually, this study is impactful because it integrates comparative efficacy, systems-level target identification, and in vivo validation within a single experimental continuum. By demonstrating clear dominance over structurally related flavonoids, the work suggests that eriodictyol’s substitution pattern confers a distinct signaling affinity rather than nonspecific cytotoxicity. Moving forward, optimization of delivery, testing in patient-derived gastric cancer models, and rational combination with PI3K-directed agents or standard chemotherapies represent logical steps toward clinical translation [97].

He et al. reported that eriodictyol has been shown to exert selective growth-suppressive effects on malignant cells while sparing the non-tumorigenic mammary epithelium, reinforcing its suitability for long-term chemopreventive intervention in breast cancer models [46]. Beyond direct cytotoxicity, the study provided evidence that eriodictyol interferes with early carcinogenic processes, significantly reducing mammary tumor incidence in a chemical carcinogenesis model, while maintaining normal body-weight trajectories over prolonged administration [46]. These findings distinguish eriodictyol from conventional anticancer agents by highlighting its capacity to modulate tumor initiation rather than solely targeting established malignancies. A central mechanistic insight of this work is the identification of circ_0007503 as a functional mediator of eriodictyol’s anticancer effects. Eriodictyol was shown to selectively downregulate circ_0007503 in breast cancer cells while leaving its expression largely unchanged in non-malignant counterparts [46]. Crucially, restoration of circ_0007503 expression partially reversed eriodictyol-mediated growth suppression, demonstrating a direct functional role for this circRNA rather than a secondary association [46]. Supporting its biological relevance, circ_0007503 levels were found to rise progressively during carcinogen-induced breast tumor development [46], identifying it as both a contributor to malignant transformation and a molecular vulnerability responsive to eriodictyol intervention.

The significance of this work lies in its identification of circRNA modulation as a previously underappreciated mechanism through which eriodictyol influences PI3K/Akt-driven breast carcinogenesis. The observation that a dietary flavanone can suppress tumor initiation by reprogramming non-coding RNA networks meaningfully broadens the mechanistic scope of flavonoid-based chemoprevention. Although the preventive design of the study limits conclusions regarding efficacy in established disease, the findings provide a compelling rationale for exploring eriodictyol in high-risk cohorts and as an adjunct to endocrine or PI3K-directed therapies. Moving forward, attention should be given to breast cancer subtype specificity, durability of circRNA regulation, and the feasibility of circ_0007503 as a predictive biomarker for flavonoid-responsive prevention strategies.

Yang et al. delivered strong mechanistic evidence that eriodictyol-derived compounds can exert pronounced anticancer effects in prostate cancer by dismantling STAT3-centered survival circuitry, a pathway widely recognized for driving disease progression and resistance to therapy [71]. In their study, eriodictyol 5-O-methyl ether (ERIO) effectively inhibited both constitutive and cytokine-stimulated STAT3 activation in androgen-independent DU145 cells as well as androgen-responsive PC-3 models. Importantly, this suppression was not confined to reduced phosphorylation alone; ERIO also prevented STAT3 nuclear translocation, thereby functionally disabling its transcriptional output [71]. By interrupting STAT3 signaling at multiple regulatory tiers, ERIO avoids the compensatory feedback loops that frequently undermine agents targeting a single signaling node.

A particularly noteworthy contribution of this work is the demonstration that STAT3 inhibition arises from coordinated disruption of upstream regulators rather than downstream interference alone. ERIO markedly attenuated phosphorylation of JAK1, JAK2, and Src kinases, establishing that STAT3 silencing reflects a broader collapse of oncogenic signaling architecture [71]. This upstream-to-downstream inhibition explains the magnitude of cytotoxicity observed and supports the classification of ERIO as a signaling-network disruptor rather than a narrow pathway antagonist. Beyond classical apoptotic execution, the study further revealed engagement of paraptosis, expanding the spectrum of regulated cell-death programs activated by ERIO. The authors propose an integrated model in which oxidative stress, mitochondrial dysfunction, and endoplasmic reticulum stress converge to drive irreversible cellular failure [71]. This dual activation of apoptotic and non-apoptotic death mechanisms is particularly relevant in advanced prostate cancer, where evasion of apoptosis represents a dominant mode of therapeutic escape.

From an expert standpoint, this work significantly advances the field by positioning eriodictyol derivatives among the few flavonoid-based agents capable of simultaneously suppressing oncogenic STAT3 signaling and activating multiple regulated death pathways [71]. The combined inhibition of upstream kinases, prevention of STAT3 nuclear function, and induction of paraptosis suggest that ERIO may overcome resistance mechanisms that limit the effectiveness of conventional STAT3 inhibitors. Future efforts should focus on validation in in vivo prostate cancer models, pharmacokinetic refinement of eriodictyol derivatives, and evaluation of combination strategies with androgen-deprivation or immunomodulatory therapies [71]. Collectively, these findings elevate eriodictyol derivatives from bioactive natural products to mechanistically substantiated anticancer leads with meaningful translational potential.

**Figure 8:**
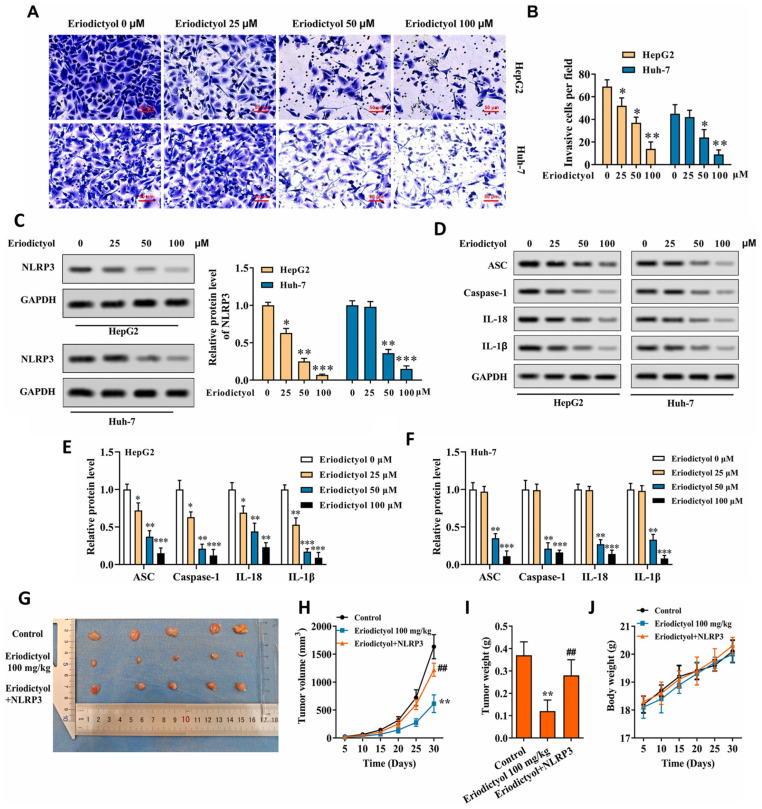
Eriodictyol restrains hepatocellular carcinoma progression by targeting NLRP3 inflammasome signaling. Eriodictyol markedly reduces the migratory and invasive capacity of hepatocellular carcinoma cells (**A**,**B**), accompanied by suppression of NLRP3 expression (**C**) and coordinated downregulation of core inflammasome components, including ASC, caspase-1, IL-1β, and IL-18 (**D**–**F**). Genetic reconstitution of NLRP3 reverses the anti-invasive effects of eriodictyol in vitro (**G**) and significantly diminishes tumor growth inhibition in xenograft models (**H**–**J**), establishing NLRP3 inflammasome inactivation as a key determinant of eriodictyol-mediated antitumor activity. (Scar bar: 50 μm). Adapted from [67]. (Statistical significance is indicated as * *p* < 0.05, ** *p* < 0.01, and *** *p* < 0.001; ## *p* < 0.01 vs. another treatment group).

Complementing these observations, Huang et al. recently identified eriodictyol as a potent inhibitor of invasion and inflammation-driven progression in hepatocellular carcinoma through direct suppression of the NLRP3 inflammasome [67]. Rather than acting primarily as an antiproliferative agent, eriodictyol markedly reduced migratory and invasive behavior in HepG2 and Huh-7 cells (Figure 8A,B), highlighting its relevance to metastatic control. This phenotypic restraint was mechanistically linked to dose-dependent downregulation of NLRP3 itself (Figure 8C), accompanied by coordinated inhibition of inflammasome components, including ASC, caspase-1, IL-1β, and IL-18 (Figure 8D–F) [67]. Crucially, the study established causality between inflammasome suppression and therapeutic response. Genetic reconstitution of NLRP3 substantially restored invasive capacity and inflammatory signaling in eriodictyol-treated cells (Figure 8G), confirming that NLRP3 functions as a determinant rather than a passive marker of response [67]. This dependency extended to in vivo outcomes, where eriodictyol significantly curtailed xenograft tumor growth and tumor burden (Figure 8H–J), effects that were largely negated upon NLRP3 overexpression [67]. Taken together, these findings position eriodictyol as a targeted modulator of inflammation-driven tumor progression, a mechanism of particular relevance in hepatocellular carcinoma, where chronic inflammasome activation fuels malignancy and therapeutic resistance [67].

From a mechanistic standpoint, this study is particularly significant because it extends eriodictyol’s anticancer relevance into the sphere of innate immune regulation, an axis increasingly viewed as therapeutically tractable in hepatocellular carcinoma [67]. By dampening NLRP3 inflammasome activity, eriodictyol interrupts a pro-tumorigenic inflammatory loop that fuels invasion, angiogenesis, and reciprocal signaling between tumor cells and the surrounding microenvironment. This mechanism is especially compelling in the context of HCC, where chronic inflammation and underlying liver disease often shape tumor biology and therapeutic response. The findings, therefore, raise the possibility that eriodictyol may be most effective in inflammation-driven HCC subsets [67]. Moving forward, validation in immunocompetent and orthotopic models will be essential, along with studies probing interactions with immune checkpoint inhibitors or anti-angiogenic agents. It will also be important to determine whether inflammasome suppression by eriodictyol translates into broader immune remodeling within the tumor microenvironment [89]. Collectively, this work positions eriodictyol as a mechanistically defined, inflammation-targeting anticancer agent with clear translational implications for liver cancer.

Although lung cancer, glioma, and gastric carcinoma provide the most detailed mechanistic frameworks, eriodictyol’s anticancer activity is not confined to these malignancies. Additional studies report induction of apoptosis and suppression of proliferation in pancreatic cancer models, where eriodictyol reduces cell viability in both time- and concentration-dependent fashions [98]. Similarly, Li et al. [25] documented inhibitory effects across colorectal, breast, pancreatic, and hepatic cancer cell lines, albeit with lineage-specific sensitivity, glioma cells showing the most pronounced response. These broader observations suggest that eriodictyol’s cytotoxic and cytostatic actions arise from modulation of conserved regulatory circuits that underpin malignant behavior, rather than from context-limited or tumor-specific mechanisms [25].

Despite this encouraging scope, important limitations remain. Many investigations rely on a limited number of cell lines, restricting insight into inter-tumoral heterogeneity. Reported effective concentrations often exceed those likely achievable through conventional oral administration, emphasizing the need for optimized delivery strategies. Furthermore, while suppression of PI3K/Akt signaling is consistently observed, downstream consequences—such as effects on EMT programs, redox balance, and cell-cycle control—are not uniformly interrogated across cancer models. Equally critical is the lack of systematic comparisons between malignant and non-malignant cells, an omission that complicates assessment of therapeutic selectivity and safety. Addressing these gaps will be essential for defining eriodictyol’s true clinical potential.

In vitro data strongly support eriodictyol as a broad-spectrum anticancer agent. Its consistent ability to impede cell viability, induce apoptosis, and inhibit migration/invasion across multiple cancer cell types, especially when tied to modulation of canonical oncogenic signaling pathways, marks it as among the more promising flavonoids currently under preclinical investigation. However, the high concentrations used, limited normal-cell comparisons, and mechanistic gaps underline the urgency of bridging to in vivo and delivery-focused studies. In particular, evidence supporting bioavailability enhancement (e.g., via nanoformulation) will likely determine whether eriodictyol can transition from in vitro promise to translational reality. Table 1 summarizes the key findings of studies investigating eriodictyol as an anticancer agent.

## 5. Eriodictyol as a Therapeutic Sensitizer and Adjuvant in Multimodal Medicine

Beyond its intrinsic anticancer activity, eriodictyol is increasingly recognized for its value as a synergistic and adjuvant agent, capable of enhancing therapeutic efficacy by reshaping cellular vulnerability rather than acting as a stand-alone cytotoxic compound [33]. Cancer progression and resistance to treatment are fueled by adaptive signaling plasticity, redox buffering, inflammatory reinforcement, and evasion of regulated cell death—processes that eriodictyol can uniquely influence simultaneously. By suppressing survival pathways (such as PI3K/Akt/mTOR and ERK signaling) [102], destabilizing redox and inflammatory homeostasis, and lowering apoptotic and ferroptotic activation thresholds, eriodictyol can sensitize malignant cells to chemotherapeutics, targeted inhibitors, and radiation-based modalities [67]. This adjuvant role fundamentally reframes eriodictyol not as a standalone cytotoxic agent, but as a biological amplifier capable of enhancing therapeutic responsiveness. Its pleiotropic yet coordinated molecular actions make it particularly well suited for incorporation into combination strategies aimed at overcoming drug resistance, lowering effective doses of conventional agents, and minimizing systemic toxicity. In this context, eriodictyol aligns naturally with advanced delivery and treatment platforms, including nanocarrier-based systems, combinatorial drug regimens, and radiotherapeutic approaches. Collectively, this framework supports the view of eriodictyol as a functional adjuvant within next-generation anticancer strategies and provides a conceptual foundation for future translational development.

**Figure 9:**
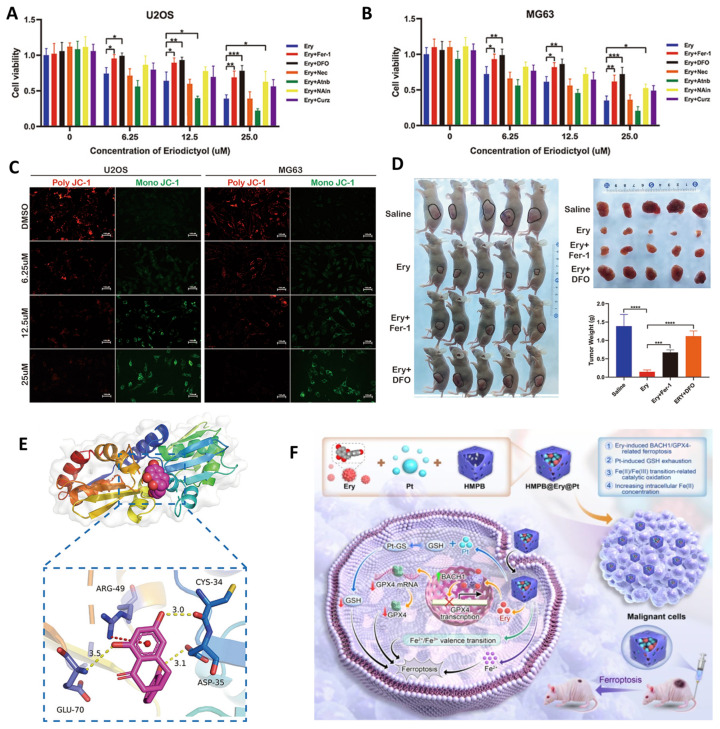
Eriodictyol induces ferroptosis and enhances chemotherapy efficacy in osteosarcoma through BACH1–GPX4 regulation and nanotherapeutic delivery. Eriodictyol reduces osteosarcoma cell viability and enhances sensitivity to ferroptosis-inducing agents in U2OS and MG63 cells (**A**,**B**), accompanied by ferroptosis-associated mitochondrial dysfunction (**C**). Combination treatment suppresses tumor growth in vivo (**D**). Molecular docking identifies BACH1 as a direct target of eriodictyol (**E**), while nanomedicine-based co-delivery of eriodictyol and cisplatin amplifies ferroptosis and antitumor efficacy through coordinated redox and iron modulation (**F**). (Scar bar: 100 μm). Adopted from [33]. (Differences between groups are denoted by * *p* < 0.05, ** *p* < 0.01, *** *p* < 0.001, and **** *p* < 0.0001).

A striking illustration of this paradigm shift is provided by a recent study in which eriodictyol was repurposed as a nanomedicine-enabled ferroptosis sensitizer. Encapsulation of eriodictyol within hollow mesoporous Prussian blue nanocubes led to a marked amplification of anticancer activity in osteosarcoma models (Figure 9A–F) [33]. Compared with free eriodictyol or single-agent treatments, the nanoformulated system achieved substantially greater suppression of tumor cell viability and heightened sensitivity to ferroptotic stress (Figure 9A,B), underscoring the critical role of delivery architecture in unlocking eriodictyol’s therapeutic potential [33]. At the cellular level, nanocarrier-assisted delivery intensified mitochondrial dysfunction and lipid peroxidation (Figure 9C), establishing ferroptosis as the dominant mode of tumor cell elimination [33].

Mechanistic analyses revealed that the nanoplatform preserved and potentiated eriodictyol’s interaction with the BACH1–GPX4 regulatory axis. Structural modeling supported direct binding of eriodictyol to BACH1 (Figure 9E), leading to transcriptional repression of GPX4. This effect was further reinforced by the nanocarrier itself, which promoted intracellular iron release and glutathione depletion, collectively creating a redox environment highly permissive to ferroptotic commitment (Figure 9F) [33]. Importantly, these coordinated molecular events translated into robust inhibition of tumor growth in vivo (Figure 9D), demonstrating that nanoformulation is not merely additive but essential for converting eriodictyol into an effective ferroptosis-directed anticancer agent [33]. Taken together, this study exemplifies how nanotechnology can transform flavonoids from modest bioactives into pathway-focused anticancer sensitizers with genuine therapeutic relevance. By integrating flavonoid-mediated transcriptional control with nanomaterial-driven metabolic stress, the work establishes a powerful paradigm for ferroptosis-based chemosensitization [33]. Future investigations should prioritize pharmacokinetic optimization, validation in orthotopic and metastatic osteosarcoma models, and exploration of whether eriodictyol-centered nanomedicines can induce immunogenic ferroptotic responses that synergize with immunotherapy.

**Figure 10:**
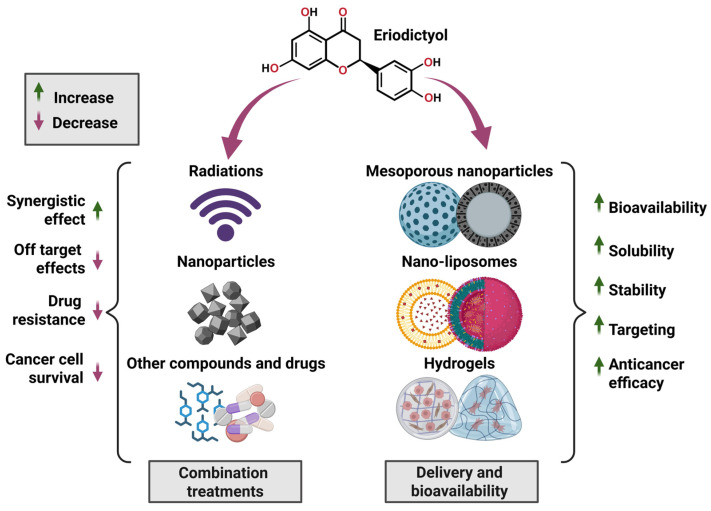
Emerging translational strategies and future research directions for eriodictyol-based anticancer therapy. Eriodictyol may be integrated with nanocarrier systems, combination treatments, and radiation-based modalities to improve bioavailability, solubility, stability, tumor targeting, and anticancer efficacy. Such approaches aim to enhance therapeutic synergy while reducing off-target effects, drug resistance, and cancer cell survival. Created in BioRender. Mumtaz, S. (2026) https://BioRender.com/opmijwq.

Figure 10 provides an integrative overview of how eriodictyol functions as a therapeutic sensitizer by enhancing synergistic interactions with conventional therapies and plant-derived compounds while simultaneously overcoming bioavailability and drug-resistance barriers through advanced delivery strategies [16,17]. Table 2 summarizes the key advantages and practical limitations of eriodictyol-based synergistic and delivery strategies, highlighting both their translational promise and the challenges that must be addressed for clinical implementation.

## 6. Advanced Delivery Strategies to Enhance the Anticancer Potential of Eriodictyol

Despite mounting evidence supporting eriodictyol as a multi-pathway anticancer flavanone, its clinical translation remains constrained by unfavorable physicochemical and pharmacokinetic properties, including limited aqueous solubility, rapid metabolism, and suboptimal tumor accumulation [106,107,108]. These limitations are characteristic of polyphenolic flavonoids and necessitate the development of advanced delivery strategies capable of preserving bioactivity while improving systemic exposure and tumor selectivity (Figure 10). Unlike earlier discussions that broadly speculated on flavonoid nanocarriers, this review emphasizes functionally validated and mechanistically justified delivery approaches for eriodictyol, with particular relevance to oncology (Figure 10). Recent studies indicate that nano-enabled delivery systems represent the most promising strategy to unlock the therapeutic potential of eriodictyol. Encapsulation within nanocarriers enhances solubility and stability, protects eriodictyol from premature degradation, and enables controlled release within the TME [106,107,108]. Importantly, nanoformulation transforms eriodictyol from a modestly bioactive compound into a pathway-directed anticancer sensitizer, as exemplified by ferroptosis-amplifying and chemosensitization platforms reported in osteosarcoma models. In these systems, nanocarriers not only improve pharmacokinetics but also actively participate in therapeutic action by modulating redox balance, iron availability, or intracellular drug accumulation [107].

Moving beyond conventional carrier systems, tumor-responsive and pathway-aligned nanoplatforms appear especially well matched to eriodictyol’s biological profile [109]. Because eriodictyol engages multiple oncogenic and stress-adaptive pathways—including PI3K/Akt/mTOR, STAT3, NLRP3 inflammasome signaling, apoptosis, and ferroptosis—delivery systems engineered to respond to tumor-specific cues such as oxidative stress, acidic microenvironments, or iron overload have the potential to amplify its activity in a biologically coherent manner [96,110]. Context-triggered release not only improves intratumoral accumulation but also limits unnecessary exposure in normal tissues, thereby sharpening pathway engagement where it is most relevant.

Although liposomal, polymer-based, and inorganic nanocarriers have demonstrated feasibility, the field is clearly moving toward multifunctional nanomedicines. In these platforms, eriodictyol is incorporated alongside chemotherapeutics or immune-modulating agents in designs that enable coordinated, rather than additive, biological effects. Such co-delivery strategies are particularly attractive in resistant tumors, as eriodictyol has been shown to lower apoptotic thresholds, sensitize cells to ferroptotic stress, and weaken kinase-driven survival programs. In this setting, nanotechnology serves not simply as a transport vehicle but as a means of reshaping tumor vulnerability landscapes to favor therapeutic response [111]. By contrast, non-nano delivery approaches—including microencapsulation, polymer matrices, or transdermal systems—remain largely unexplored for eriodictyol in oncology and have so far been considered mainly in anti-inflammatory or antioxidant contexts. As a result, future translational efforts should focus on tumor-directed nanodelivery, rigorous pharmacokinetic optimization, and validation in clinically relevant models, including orthotopic and patient-derived tumor systems.

In summary, advanced delivery strategies should be viewed not as optional enhancements but as central determinants of eriodictyol’s anticancer potential. The integration of rational nanodesign with eriodictyol’s multi-target pharmacology offers a credible path toward translating experimental efficacy into clinically meaningful outcomes, positioning eriodictyol as a realistic component of precision oncology rather than a purely exploratory natural product.

## 7. Limitations and Challenges

Despite growing experimental support for eriodictyol as a multi-target anticancer flavanone, several obstacles currently limit its translational progression. A primary challenge is its physicochemical instability. As a polyphenolic molecule, eriodictyol is susceptible to degradation under physiological pH variation, oxidative stress, and thermal exposure, which can reduce effective drug levels at tumor sites and necessitate protective formulation strategies.

A second major limitation concerns tumor-selective delivery. Although nanocarrier-based systems can enhance solubility and circulation time, their in vivo distribution remains highly context-dependent. Off-target accumulation in clearance organs and uptake by the reticuloendothelial system may compromise safety and narrow the therapeutic window, particularly for a compound whose anticancer activity depends on finely balanced redox and apoptotic modulation. In addition, controlled and tumor-responsive release remains technically challenging. The heterogeneous nature of the tumor microenvironment—characterized by variable pH, oxygen tension, and redox status—can unpredictably affect carrier stability and intracellular drug availability, potentially limiting pathway engagement.

Although mechanistic insights into eriodictyol’s anticancer activity are substantial, it is important to note that the majority of available evidence derives from in vitro cell-based models. While these systems are essential for pathway-level resolution, they do not fully capture the complexity of tumor–host interactions, pharmacokinetics, immune modulation, or toxicity profiles encountered in vivo. At present, in vivo studies remain relatively limited and are largely confined to xenograft models, and robust clinical data are essentially absent. This imbalance restricts direct extrapolation to patient settings and underscores the need for systematic validation in animal models with clinically relevant dosing, followed by carefully designed translational and early-phase clinical studies.

An additional limitation arises from methodological heterogeneity across published studies. Investigations of eriodictyol employ diverse cancer models, exposure durations, concentration ranges, and experimental readouts, which complicates direct comparison of effect magnitude or potency across tumor types. Such variability limits the ability to define a single quantitative benchmark for anticancer strength. However, despite these differences, a consistent qualitative pattern emerges across independent studies, with recurrent involvement of shared signaling pathways governing proliferation, apoptosis, inflammation, and stress adaptation. This convergence supports the robustness of the underlying mechanisms while underscoring the need for future studies employing standardized models, harmonized dosing strategies, and comparable endpoints to enable more rigorous cross-study evaluation.

Beyond mechanistic efficacy, several practical aspects of eriodictyol pharmacology remain insufficiently resolved. Available data on absorption, metabolic conversion, tissue distribution, effective dosing ranges, and long-term safety are fragmentary and largely derived from short-term preclinical studies. Although existing in vivo reports suggest limited acute toxicity, systematic pharmacokinetic analyses and chronic exposure studies are lacking, restricting confident extrapolation to clinical settings. Emerging nanocarrier systems and combination strategies offer promising routes to overcome solubility and bioavailability constraints; however, these approaches remain predominantly preclinical and require rigorous validation in clinically relevant models. At present, such delivery platforms should be regarded as exploratory tools to enhance biological understanding rather than mature therapeutic solutions. Addressing these pharmacological and translational gaps will be essential for determining whether eriodictyol can progress beyond experimental promise toward realistic clinical utility.

Finally, manufacturing scalability and regulatory feasibility represent non-trivial barriers. Many advanced delivery platforms proposed for eriodictyol rely on complex fabrication and surface modification steps, raising concerns related to reproducibility, long-term stability, and regulatory approval. Taken together, these challenges emphasize that successful translation of eriodictyol will require coordinated optimization of pharmacology, delivery engineering, and translational feasibility, rather than reliance on biological activity alone.

It should also be noted that the relative hierarchy of mechanisms attributed to eriodictyol remains unresolved. Pathways such as PI3K/Akt/mTOR inhibition, apoptosis, redox modulation, and ferroptosis are likely interconnected and context-dependent rather than uniformly dominant across cancer models. As with any literature-based review, potential publication bias must be considered, as negative or neutral findings are less frequently reported.

## 8. Future Perspectives and Translational Outlook

The body of evidence assembled in this review moves eriodictyol beyond the category of a generically “bioactive” flavanone and toward recognition as a systems-level modulator of cancer cell behavior. Eriodictyol redirects malignant cell fate by targeting multiple pathways, including cell-cycle control, apoptosis, oncogenic survival signals—like PI3K/Akt/mTOR and STAT3—and inflammation pathways such as NLRP3. It also affects redox balance and iron homeostasis, influencing ferroptotic vulnerability. These combined actions suggest eriodictyol works at an integrated signaling level, matching the complex nature of cancer progression and resistance. The most important implication of this mechanistic breadth is strategic: future progress will not come from simply adding more in vitro cytotoxicity reports, but from engineering context-specific eriodictyol interventions that exploit defined vulnerabilities (apoptosis resistance, ferroptosis fragility, kinase-addiction, inflammatory microenvironments, and therapy-induced adaptation) in clinically relevant models [112,113].

A central priority is to elevate eriodictyol research from “single-agent activity” to precision deployment [114,115]. Many tumors that appear only modestly sensitive to free eriodictyol in standard assays may become highly responsive when eriodictyol is used as a sensitizer rather than a primary cytotoxic, lowering the threshold for apoptosis, collapsing survival signaling, or disabling adaptive stress buffering [116,117]. This requires future studies to adopt a design logic aligned with modern oncology: (i) define the dominant resistance mechanism in a given tumor context (e.g., anti-apoptotic BCL-2 family buffering, hyperactive PI3K/Akt/mTOR, STAT3-driven survival, inflammasome-linked angiogenesis, or GPX4-dependent ferroptosis protection), and (ii) map eriodictyol’s engagement of that mechanism using causality-resolving experiments (genetic rescue, pathway reactivation, or pharmacologic antagonism). Such approaches will yield results that are both mechanistically convincing and clinically understandable [112,113].

A second frontier is delivery-enabled pharmacology, in which eriodictyol’s challenges, poor solubility, limited stability, rapid clearance, and inadequate tumor accumulation are more engineering challenges than biological flaws [32]. Nanotechnology should be regarded as essential to mechanism-driven therapy rather than optional. Recent eriodictyol nanomedicine studies show how delivery platforms can target the compound’s activity by controlling intracellular exposure, enriching tumor tissue, and adding stressors like iron dysregulation, redox imbalance, and glutathione depletion [33,108,118]. Future platforms should therefore prioritize pathway-coupled design, where the carrier is selected not only for pharmacokinetics but also for its ability to synergize with eriodictyol’s mechanistic signature (e.g., ROS-responsive release in oxidative tumors; pH-triggered release in acidic microenvironments; iron-enabled carriers for ferroptosis priming) [119,120].

A third opportunity is a combination logic that is mechanistically disciplined. The field should move beyond empirical pairing and instead use eriodictyol to solve specific therapeutic problems: overcoming acquired resistance to kinase inhibitors, restoring apoptotic competence to chemotherapy-refractory tumors, suppressing pro-metastatic inflammatory signaling, or reshaping oxidative homeostasis to re-enable treatment response [121]. Combinations should be rationally tiered into: (i) signal-axis combinations (eriodictyol with inhibitors targeting PI3K/Akt/mTOR, HER2/EGFR kinases, STAT3, or MEK/ERK nodes when relevant), (ii) death-pathway combinations (eriodictyol with agents that trigger extrinsic apoptosis or dismantle anti-apoptotic buffering) [121], and (iii) ferroptosis-enhancing combinations (eriodictyol with therapies that deplete glutathione, disrupt lipid peroxide detoxification, or increase labile iron) [99,122].

A fourth, growing area of focus is testing eriodictyol in models that more closely mimic human disease biology for representing the complexity of TME interactions, immune responses, and metastatic progression [123]. Meaningful progress requires systematic testing in patient-derived organoids, co-culture systems with immune and stromal elements, orthotopic tumor models, and tumors reflecting treatment histories. These models are crucial for evaluating angiogenesis regulation, inflammasome suppression, resistance, and ferroptosis, which are influenced by spatial, metabolic, and immunological context, not just tumor cell behavior.

Within this forward-looking landscape, Figure 10 serves as a translational roadmap that distills the central conclusions of this review into actionable research directions. Figure 10 highlights two interconnected pillars likely to shape the next phase of eriodictyol development [123]. First, it highlights rational combination strategies, pairing eriodictyol with other agents to boost efficacy and reduce resistance. Second, it emphasizes delivery-focused optimization, such as mesoporous nanoparticles, nanoliposomes, and hydrogels, to improve solubility, stability, tumor targeting, and pharmacological effects [16].

Despite growing experimental evidence supporting the biological activities of eriodictyol, the precise molecular targets and context-dependent mechanisms underlying its actions remain incompletely characterized. In the future, the integration of advanced target discovery strategies—such as multi-omics analyses and chemical probe-based approaches—may provide deeper insights into the molecular interactions of natural products [124]. In addition, emerging probe technologies, including proteolysis-targeting chimera (PROTAC)-based systems developed for target deconvolution, represent promising methodological tools that could be explored to further elucidate the intracellular targets and signaling networks associated with bioactive small molecules [125]. Looking ahead, the field should focus on key milestones to assess eriodictyol’s clinical potential. These include detailed pharmacokinetic studies of free and formulated eriodictyol, demonstrating mechanism-backed efficacy in relevant models, supported by genetic or pharmacological evidence; developing combination therapies based on resistance biology that are truly synergistic; and creating scalable, reproducible manufacturing with clear regulatory paths. Achieving these would elevate eriodictyol from a promising natural compound to a strategic element in modern oncology, capable of disrupting interconnected cancer survival programs.

## 9. Conclusions

This review summarizes current evidence identifying eriodictyol as a biologically relevant flavanone in anticancer research. Rather than targeting a single pathway, eriodictyol acts as a multi-pathway modulator that limits tumor progression across diverse cancer models. It suppresses cell proliferation by disrupting cyclin–CDK activity, inhibiting major survival pathways such as PI3K/Akt/mTOR, and inducing cell-cycle arrest. These effects are accompanied by activation of regulated cell-death mechanisms, including apoptosis, through mitochondrial disruption, caspase activation, and death-receptor signaling. Eriodictyol also alters tumor redox balance, increases sensitivity to oxidative stress, and shows emerging roles in inflammation-related signaling and ferroptosis regulation. Importantly, advances in delivery strategies, particularly nano-based systems, may overcome pharmacokinetic limitations and enhance therapeutic efficacy. Collectively, these findings support eriodictyol as a promising multi-functional candidate for future anticancer development and rational combination therapies.

## Figures and Tables

**Table 1:** Overview of anticancer effects of eriodictyol. (arrows “↑” indicate increase or activation, “↓” indicate decrease or inhibition).

Type	Targeted Pathway	Eriodictyol Concentration	Main Findings	Ref.
Lung cancer (in vitro)	PI3K/Akt/mTOR	IC_50_ ≈ 50 µM (A549); ≈95 µM (FR2)	↑ Apoptosis, ↑ Bax, ↑ cleaved caspase-3, ↑ G2/M arrest, ↑ ΔΨm loss; ↓ Proliferation, ↓ Bcl-2, ↓ Akt, ↓ mTOR, ↓ PI3K/Akt/mTOR signaling	[27]
Glioma (in vitro & in vivo)	PI3K/Akt/NF-κB	25, 50, and 100 µM	↑ Apoptosis, ↑ Bax, ↑ cleaved PARP; ↓ Proliferation, ↓ migration, ↓ invasion, ↓ Akt, ↓ NF-κB signaling; ↓ Xenograft tumor growth with minimal toxicity	[25]
Nasopharyngeal carcinoma	MEK/ERK	10, 20, and 40 µM	↑ Autophagy (↑ LC3B), ↑ apoptosis; ↓ Proliferation, ↓ migration, ↓ invasion, ↓ MEK/ERK signaling	[47]
Glioma (autophagy)	PI3K/Akt; autophagy axis	25, 50, 75, and 100 mM	↑ Apoptosis, ↑ Bax, ↑ LC3B, ↑ Beclin-1; ↓ Cell viability, ↓ p-PI3K, ↓ p-Akt	[99]
Glioblastoma (EMT)	P38 MAPK/GSK-3β/ZEB1; EMT pathway	5–100 µM (in vitro); 200 mg kg^−1^ (in vivo)	↑ E-cadherin, ↑ epithelial phenotype; ↓ Migration, ↓ invasion, ↓ EMT-like transition, ↓ N-cadherin, ↓ MMP-2, ↓ ZEB1; ↓ p-P38 MAPK, ↓ p-GSK-3β (Tyr216); ↓ EMT markers in vivo with favorable safety vs. temozolomide	[68]
Colorectal cancer	TSTA3/fucosylation	0–400 µM	↑ Apoptosis; ↓ Proliferation, ↓ clonogenicity, ↓ migration, ↓ invasion, ↓ EMT, ↓ TSTA3-dependent fucosylation	[100]
Gastric cancer	PI3K/Akt	50, 100, 150 µM	↑ Apoptosis, ↑ cleaved caspase-3, ↑ G2/M arrest; ↓ Proliferation, ↓ PI3K/Akt phosphorylation; ↓ Xenograft tumor growth with low systemic toxicity	[26]
Breast cancer (chemoprevention)	circ_0007503–PI3K/Akt	10, 20, and 40 µM	↑ Apoptosis; ↓ Cell viability, ↓ carcinogen-induced transformation, ↓ circ_0007503 expression, ↓ PI3K/Akt signaling; ↓ Mammary tumor incidence in vivo	[46]
Multi-cancer (TNFR1-dependent)	TNFR1/FADD/TRADD; extrinsic apoptosis	25–200 µM; 60 mg kg^−1^ (in vivo)	↑ TNFR1 (cancer-specific), ↑ DISC formation, ↑ caspase-8/7 activation, ↑ PARP cleavage, ↑ G2/M arrest; ↓ NF-κB signaling, ↓ tumor growth, ↓ lung metastasis in vivo	[48]
Hepatocellular carcinoma	NLRP3 inflammasome	25–100 µM	↓ Migration, ↓ invasion, ↓ angiogenesis; ↓ NLRP3, ↓ ASC, ↓ caspase-1, ↓ IL-1β, ↓ IL-18; ↓ Tumor growth in vivo (reversed by NLRP3 overexpression)	[67]
Oral squamous cell carcinoma	ROS–MAPK/STAT3	30 and 40 µM	↑ ROS, ↑ apoptosis, ↑ caspase activation; ↓ Proliferation, ↓ MAPK signaling, ↓ STAT3 activation	[101]
Skin cells (UVA-induced photodamage; non-cancer)	ROS–MAPK (ERK/JNK/p38); MMP-1 axis	5–40 µM	↓ ROS generation, ↓ LDH leakage, ↓ inflammatory cytokines (IL-1β, IL-6, TNF-α, COX-2), ↓ MMP-1 expression, ↓ p-ERK, ↓ p-JNK, ↓ p-p38; ↑ SOD activity, ↑ TIMP-1, ↑ collagen I (COL-1)	[75]
Osteosarcoma (nano-enabled)	BACH1–GPX4; ferroptosis	IC_50_ ≈ 26–27 µM (free); nano-co-delivery in vivo	↑ Ferroptosis, ↑ lipid peroxidation, ↑ Fe^2+^ accumulation, ↑ mitochondrial damage; ↓ GPX4, ↓ GSH; Nano-co-delivery: ↑ cisplatin chemosensitivity, ↓ tumor growth with minimal organ toxicity	[33]
Ovarian cancer (in vitro & in vivo)	Nrf2/HO-1/NQO1; ferroptosis; mitochondrial dysfunction	IC_50_ (CaoV3): 229.7 µM (24 h), 38.4 µM (48 h); IC_50_ (A2780): 248.3 µM (24 h), 64.3 µM (48 h)	↑ Apoptosis, ↑ ROS, ↑ Fe^2+^ accumulation, ↑ cytochrome-c release, ↑ mitochondrial dysfunction; ↓ Cell viability, ↓ JC-1 ratio, ↓ GSH, ↓ SLC7A11, ↓ GPX4, ↓ Nrf2 phosphorylation, ↓ HO-1/NQO1 signaling; ↓ Tumor growth	[51]
Liver injury model (mechanistic relevance to cancer)	PI3K/Akt; oxidative stress–apoptosis axis	10–40 mg kg^−1^ (in vivo, mice)	↑ PI3K/Akt activation, ↑ antioxidant defenses (↑ SOD, ↑ GSH), ↑ Bcl-xl; ↓ Oxidative stress (↓ MDA), ↓ inflammatory cytokines (↓ TNF-α, ↓ IL-1β, ↓ IL-6), ↓ hepatocyte apoptosis, ↓ Bax, ↓ caspase activation; ↓ Liver injury markers and tissue damage	[102]
Acute pancreatitis	ZBP1-dependent PANoptosis (ZBP1/NLRP3/RIPK1/Caspase-8/MLKL/GSDMD axis)	5–10 mg kg^−1^ (in vivo, mice); 5–50 µM (in vitro, acinar cells)	↓ ZBP1 expression and activity, ↓ PANoptosis signaling (↓ NLRP3, ↓ pRIPK1, ↓ cleaved Caspase-8, ↓ GSDMD-N, ↓ pMLKL); ↓ pancreatic inflammation and necrosis, ↓ IL-1β and ↓ IL-18 release, ↓ LDH, ↓ lipase and amylase; ↑ acinar cell survival; minimal effect on cytosolic mtDNA	[103]
Premature ovarian failure (relevance to cancer survivorship)	PI3K/Akt/NF-κB; macrophage-mediated inflammation	20–80 mg kg^−1^ (in vivo, mice); 12.5–25 µM (in vitro co-culture)	↑ Ovarian function and reserve (↑ estrous cyclicity, ↑ ovarian index, ↑ follicle count), ↑ serum E2 and ↑ AMH; ↓ FSH; ↓ PI3K/Akt/NF-κB phosphorylation, ↓ ovarian inflammation; ↓ macrophage-mediated damage to granulosa cells; overall ↓ chemotherapy-induced ovarian dysfunction	[104]
Non-alcoholic fatty liver disease (mechanistic relevance to cancer)	UBA52-mediated autophagy; Nrf2/HO-1; NF-κB; oxidative stress	50–100 mg kg^−1^ (in vivo, HFD & db/db mice); 50–100 µM (in vitro, HepG2)	↑ Autophagy activation (↑ LC3-II/I, ↓ p62), ↑ Nrf2 nuclear translocation, ↑ HO-1 expression; ↓ UBA52, ↓ oxidative stress (↓ ROS, ↓ MDA, ↑ SOD), ↓ inflammatory cytokines (↓ TNF-α, ↓ IL-6), ↓ NF-κB activation; ↓ hepatic lipid accumulation, ↓ TG/TC, ↓ ALT/AST; improved liver histology and function	[105]

**Table 2:** Advantages and limitations of eriodictyol-based synergistic and delivery strategies in anticancer applications.

Strategy	Key Advantages	Major Limitations/Challenges
Combination with chemotherapeutic drugs	Enhances anticancer efficacy via multi-pathway targeting (PI3K/Akt, MAPK, STAT3); sensitizes resistant cancer cells; reduces required dose of cytotoxic drugs; mitigates multidrug resistance	Risk of pharmacokinetic mismatch; potential drug–drug interactions; optimization of dosing schedules required; limited clinical validation
Combination with radiotherapy	Acts as a radiosensitizer by modulating ROS and mitochondrial stress; improves tumor control while potentially protecting normal tissues through redox modulation	Narrow therapeutic window; dual antioxidant/pro-oxidant behavior may complicate timing; limited mechanistic clarity in vivo design
Nanoparticles (polymeric/inorganic)	Improves solubility, stability, and tumor accumulation; enables passive targeting via EPR effect; protects eriodictyol from premature degradation	Batch-to-batch variability; possible long-term nanotoxicity; scale-up and regulatory complexity
Mesoporous nanoparticles	High loading capacity; controlled and stimuli-responsive release; tunable pore size for precision delivery	Premature drug leakage; structural instability in physiological fluids; complex synthesis
Nano-liposomes	Biocompatible and clinically familiar platform; enhances circulation time; improves intracellular uptake	Limited drug loading efficiency; potential leakage during storage; stability concerns
Hydrogels	Localized and sustained release; reduced systemic toxicity; suitable for post-surgical or localized tumors	Limited applicability for metastatic disease; diffusion-controlled release may be inconsistent
Other nanocarriers (hybrids, biomimetic systems)	Enhanced targeting and immune evasion; improved bioavailability and tumor specificity	High production cost; complex characterization; translational barriers
Combination with other flavonoids or plant-derived compounds	Produces complementary or synergistic anticancer effects via pathway convergence (e.g., PI3K/Akt, NF-κB, MAPK); enhances redox imbalance in cancer cells; reduces toxicity through dose-sharing; supports multitarget phytochemical therapy	High compositional variability; unpredictable synergy or antagonism; challenges in standardization and reproducibility; limited pharmacokinetic and clinical evidence
Overall synergistic strategy	Simultaneous reduction in cancer cell survival, drug resistance, and off-target effects; aligns with precision oncology and multimodal therapy	Lack of standardized formulations; insufficient long-term safety data; need for clinical trials

## Data Availability

No new data were created or analyzed in this study. Data sharing is not applicable to this article.

## Abbreviations

The following abbreviations are used in this manuscript:

CDK	Cyclin-dependent kinase
EMT	Epithelial–mesenchymal transition
MMP	Matrix metalloproteinase
VEGF	Vascular endothelial growth factor
ROS	Reactive oxygen species
MAPK	Mitogen-activated protein kinase
pRb	Retinoblastoma Protein
FADD	Fas-Associated protein with Death Domain
APAF	Apoptotic Protease Activating Factor
SMAC	Second Mitochondria-derived Activator of Caspases
VRAP	VEGF Receptor-Associated Protein
ERK	Extracellular Signal-Regulated Kinase
JNK	c-Jun N-terminal Kinase
NSCLC	Non-Small-Cell Lung Cancer
TME	Tumor microenvironment
